# Gut microbiota resilience in horse athletes following holidays out to pasture

**DOI:** 10.1038/s41598-021-84497-y

**Published:** 2021-03-03

**Authors:** Núria Mach, Léa Lansade, David Bars-Cortina, Sophie Dhorne-Pollet, Aline Foury, Marie-Pierre Moisan, Alice Ruet

**Affiliations:** 1 Animal Genetic and Integrative Biology, University of Paris-Saclay, INRAE, AGroParisTech, 78350 Jouy-en-Josas, France; 2 PRC, INRAE, CNRS, IFCE, University of Tours, 37380 Nouzilly, France; 3 Medicine Department, University of Lleida, 25198 Lleida, Spain; 4 University of Bordeaux, INRAE, NutriNeuro UMR 1286, 33076 Bordeaux, France

**Keywords:** Microbiology, Microbial communities, Microbiome

## Abstract

Elite horse athletes that live in individual boxes and train and compete for hours experience long-term physical and mental stress that compromises animal welfare and alters the gut microbiota. We therefore assessed if a temporary period out to pasture with conspecifics could improve animal welfare and in turn, favorably affect intestinal microbiota composition. A total of 27 athletes were monitored before and after a period of 1.5 months out to pasture, and their fecal microbiota and behavior profiles were compared to those of 18 horses kept in individual boxes. The overall diversity and microbiota composition of pasture and control individuals were temporally similar, suggesting resilience to environmental challenges. However, pasture exposure induced an increase in *Ruminococcus* and *Coprococcus* that lasted 1-month after the return to individual boxes, which may have promoted beneficial effects on health and welfare. Associations between the gut microbiota composition and behavior indicating poor welfare were established. Furthermore, withdrawn behavior was associated with the relative abundances of *Lachnospiraceae* AC2044 group and Clostridiales family XIII. Both accommodate a large part of butyrate-producing bacterial genera. While we cannot infer causality within this study, arguably, these findings suggest that management practices maintained over a longer period of time may moderate the behavior link to the gut ecosystem beyond its resilience potential.

## Introduction

The gut microbiota is characterized by the capacity to be resilient when facing discrete short-term events such as acute bacterial and viral infections, antibiotic treatment or nutrient scarcity^1^. The term resilience describes the amount of stress that a system can tolerate before its current state shifts towards a new equilibrium that potentially has different functions^1^. Gut microbiota resilience is ensured through competition for resources, bactericidal activity, metabolic inhibition, biofilm formation, quorum sensing, quenching disruption of signaling molecules and spatial occlusion^1,2^. Moreover, the host actively contributes to the resilience potential of the gut microbiota by releasing antimicrobial components, producing a specialized mucus layer or by controlling the physiochemical properties of the gastrointestinal tract^1^. Microbiota resilience also has a dark side. The acquisition of a microbiota composition that has a high resilience potential and that is associated with a fitness loss of the hologenome (the collective genome of host and microbiome) may contribute to host health and welfare and be a significant obstacle in effective dietary and management interventions.

Relatively little is known about the context-dependent mechanisms that control resilience and the dynamics of gut microbiota in elite horse athletes with welfare impairment. Elite athlete horses predominantly live in individual loose boxes, a safe environment that can deteriorate the animal’s welfare^3^. Indeed, this housing system prevents animals from performing natural behaviors, which is one of the welfare concerns stated by the five freedoms framework^4^. Welfare refers to the positive mental and physical state associated with the satisfaction of physiological and behavioral needs of the individual^5^ and it depends on the subjective experience that the animals have in their environment^3^. The study of behavior is a sensitive tool to assess the animal welfare and hence the individual responses towards environmental challenges^3,6,7^.

Beyond the decline of welfare state^3^, our group has demonstrated that elite horses permanently living in individual boxes are subject to modifications of gut microbial communities^8^, illustrating the link between animal welfare and the gut microbiota. To describe the later relationship, the term “microbiota-gut-brain axis” has been coined^9^. Established pathways of communication include the central nervous system, the enteric nervous system, the neuroendocrine system and the immune system^10^. In horses, the role of gut microbiota on behavioral changes has been posited by Destrez et al*.*^11^, who observed that the concentration of amylolytic bacteria and the abundance of *Succinivibrionaceae* positively correlated to bowing (considered as an alert or alarm type of behavior) following a low fiber but high starch diet. In ponies fed high-starch diets, increased behavioral reactivity was related to changes in gut microbiota, suggesting that the composition of gut microbiota and behavior are strictly intertwined and influence each other^12^. Perry et al*.*^13^ did not measure gut-brain interactions but reported reductions in Proteobacteria in the equine cecal microbiota following travel stress and disruption in the sequence of the normal daily routine.

In humans, the imbalance in the microbiota-gut-brain axis has been linked to the pathogenesis of mental illnesses such as anxiety disorders^14,15^ and depression^16^. Enteric short-chain fatty acids (SCFAs), the major class of metabolites produced from bacterial fermentation of non-digestible carbohydrates, are widely thought to mediate the relationship between the gut microbiota and the brain. Neurotransmitters such as ɣ-amino butyric acid (GABA), dopamine and serotonin, stress hormones and immune system modulators are also thought to play at least a partial role in this molecular interchange^17,18^.

The role that gut microbiota plays in behavioral expressions of horses facing long-term exposition of physical and mental stress still needs to be explored. Additionally, data related to the resilience and dynamics of the fecal microbiota as a proxy for the gastrointestinal microbiome in chronically-stressed horses are scant. While formal proof is missing in horses, a prevailing hypothesis is that the impact of the long-term physical and emotional stress in elite athletes individually housed could be alleviated by allowing individuals to benefit from a temporary period in a more natural environment, such as out to pasture with conspecifics^19^. In turn, this sudden and single short-term exposition to a more natural environment could be beneficial for the gut microbiota (*e.g.*, increasing diversity, reducing abundance of proinflammatory taxa and rare pathobionts and increasing abundance of putatively beneficial taxa). To this aim, a group of horses living in individual boxes throughout the year was monitored before and after a period of 1.5 months on pasture and compared to a group of individually housed horses that had not been released to pasture. We then assessed if the microbiota shifts in the pasture group lasted at least 3 months after the return to individual boxes. Moreover, by jointly characterizing the composition of gut microbiota and the behavioral indicators of poor welfare, we aimed at providing a functional readout of microbial activity and improve our understanding of the gut-brain axis in athletes.

## Results

### Cohort characteristics

**Figure 1:**
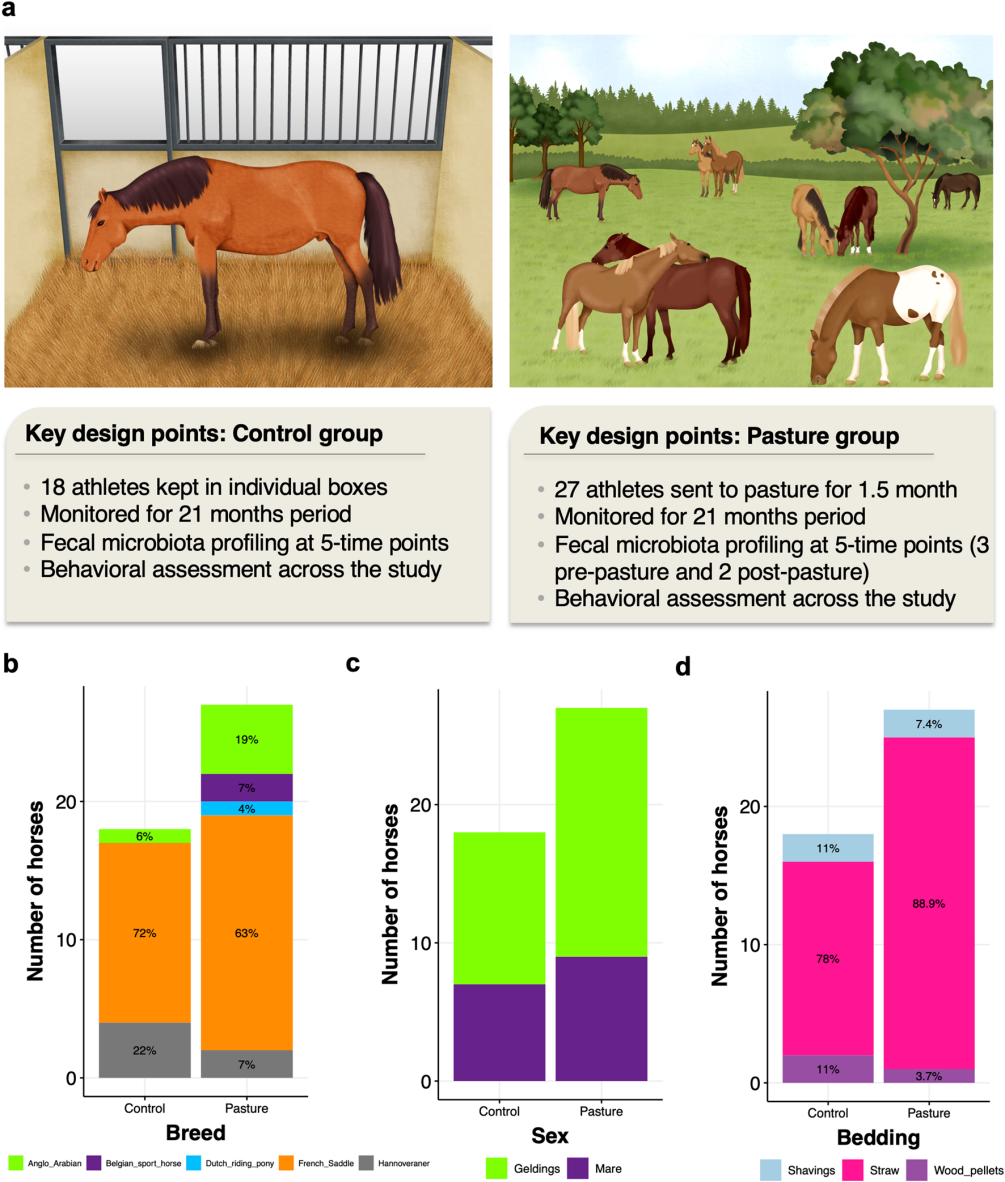
Description of the cohort. (**a**) Key points of the experimental design; (**b**) bar plot representing the number of individuals ascribed to each breed; (**c**) bar plot representing the number of individuals ascribed to each sex; (**d**) bar plot representing the number of individuals housed in straw bedding material, shavings or wood pellets. Written permission for publication of the drawing in the right panel of figure (**a**) has been given. The left pane of figure (**a**) has been obtained from Mach et al.^8^ and used according to terms of a Creative Commons Attribution 4.0. License: http://creativecommons.org/licenses/by/4.0/. No changes were made. Plots b-d were produced in R software https://www.r-project.org, using the version 4.0.2.

We selected 45 sport horses that lived in individual boxes at the same riding school and shared similar environment (Supplementary Table S1). Individuals were split into two categories based on whether they underwent a short period of 1.5 months on pasture or not (Fig. 1a). The pasture horses (*n* = 27; 17 geldings and 10 mares) were monitored three times before their release to pasture (baseline time points: T1–T3), and 1 and 3 months after their return to the box (T4 and T5, respectively). The control group consisted of 18 horses (11 geldings and 7 mares) kept in individual boxes along the study and monitored from T1 to T5. The two groups were matched regarding breed (Fig. 1b), sex (Fig. 1c) and bedding material (Fig. 1d). Overall, both groups were predominantly composed of “Selle Français” geldings with an average age of 10 ± 3.4 years (mean ± SD). Around 80% were kept in straw bedding instead of shavings or wood pellets.

### Behavior assessment

**Figure 2:**
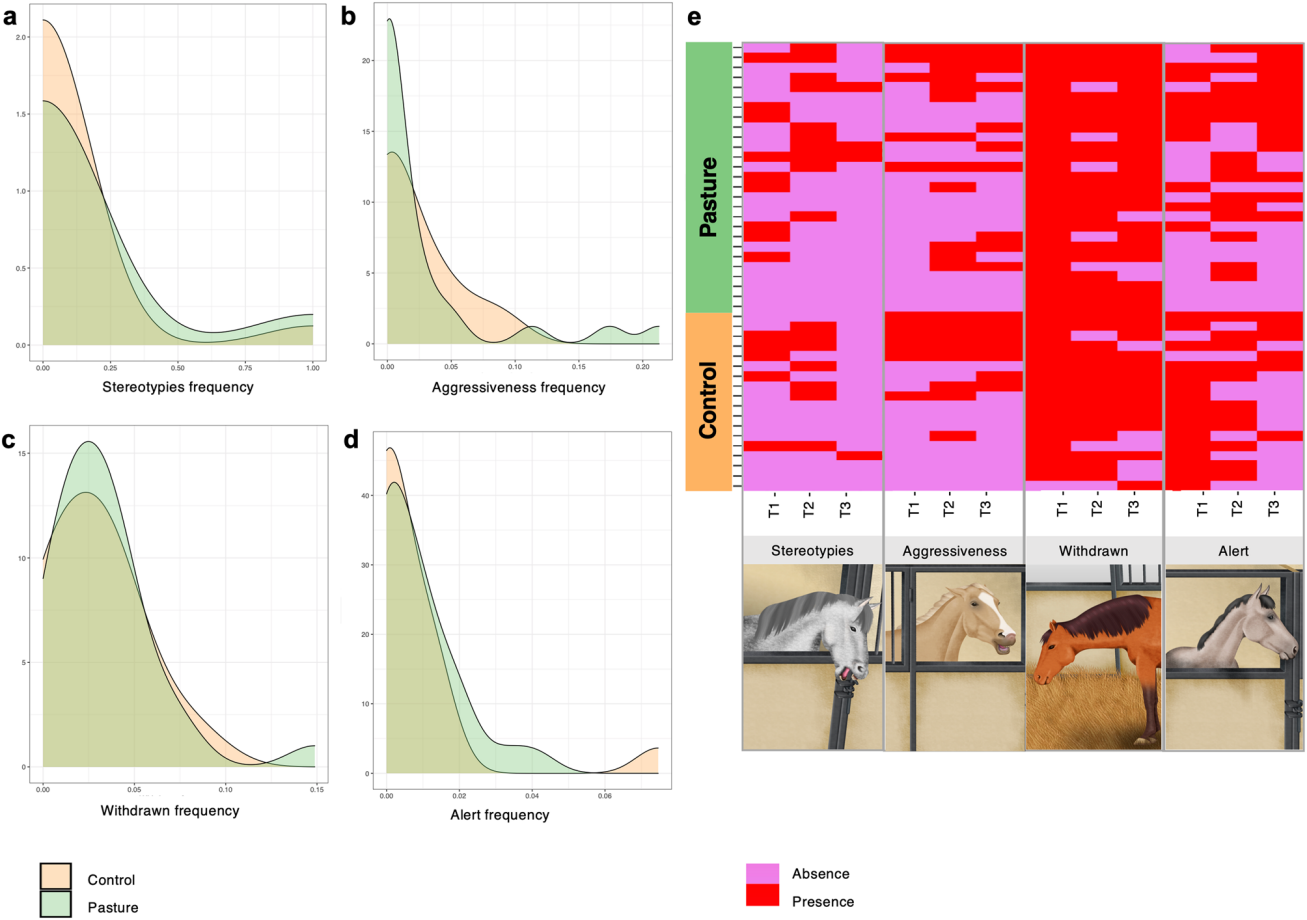
Frequency of behaviors related to welfare impairment in each of the individuals at baseline (from T1 to T3). (**a**) Density plot showing the frequency of scans of stereotypies in pasture and control groups; (**b**) density plot showing the frequency of scans of aggressiveness behavior towards humans in pasture and control groups; (**c**) density plot showing the frequency of scans of unresponsiveness to the environment in pasture and control groups; and (**d**) density plot showing the frequency of scans of hypervigilance or alert behavior in pasture and control groups; (**e**) matrix showing the presence or absence of the behavioral indicator detected in our cohort before the pasture release (from T1 to T3). Each entry in the matrix indicates the presence or absence of each behavioral indicator in each individual and time point. Individuals are grouped by treatment (pasture in green and control in orange). The drawings corresponding to the stereotypy and withdrawn behaviors have been obtained from Mach et al.^8^ and used according to terms of a Creative Commons Attribution 4.0. License: http://creativecommons.org/licenses/by/4.0/. No changes were made. Written permission for publication of the drawings corresponding to aggressiveness and alert behaviors in the figure has obtained. All plots were produced in R software https://www.r-project.org, using the version 4.0.2.

The expression of the four non-invasive behavioral indicators mirroring welfare decline^3,20,21^, namely stereotypies, aggressive behaviors towards humans, the withdrawn posture reflecting unresponsiveness to the environment, and the alert/alarm posture indicating hypervigilance were also balanced between groups before going out to pasture (*p* > 0.05; Fig. 2a–d). At baseline time points, the expression of both oral and locomotion-related stereotypies was observed in 60% of the individuals at least once regardless of the group, whilst 53% of the individuals were experiencing aggressiveness towards humans at least one time in the same period (Fig. 2e). The so-called withdrawn posture was observed in 100% of our individuals once or more, whereas hypervigilance was observed in 88% of our horses at least once (Fig. 2e).

### Dry matter intake and average energy and macronutrient intakes between groups

The individual average daily concentrate intake, the total dry matter intake (DMI) and the intake of macronutrients along the experiment are shown in the supplementary Table S2. The average daily concentrate intake was 2.71 ± 0.458 kg of dry matter (DM), total hay intake was 8.3 ± 0.13 kg (DM/day) and total DMI was 10.81 ± 0.458 kg. The daily average net energy intake from concentrate was 5.85 ± 0.990 Mcal (on DM basis), the percentage of crude protein in the diet ranged from 10.4 to 11.6% of DM intake whereas the percentage of fiber intake in the diet varied from 26.73 to 34.10%, irrespective of the group.

A mixed effect model analysis of variance was applied to quantify differences of total DMI per day between groups and its stability across time series. The total DMI was not affected by group as intended but intakes were lower at T4 and T5 (*p* < 0.05) relative to T1 (Supplementary Fig. S1a). Specifically, T4 and T5 were associated with a decrement of 0.612 kg/day of total DMI. This was the result of decreased amounts of concentrate offered from T4 onwards in some horses that had become overweight (Supplementary Table S2). Group and time factors accounted for small variation in the total DMI (marginal *R*^2^_*m*_ = 0.2797) and the far majority of variation originated from subject (conditional *R*^2^_*c*_ = 0.9019). As anticipated, intra-individual DMI remained highly stable between the measured time points (intraclass correlation coefficient (ICC) = 0.864). Given that the estimated forage intake was similar for all individuals, irrespective of the group and time points profiled, a comparable statistical trend was observed in daily concentrate (Supplementary Fig. S1b) and macronutrients intake (Supplementary Fig. S1c,d).

### Medical treatments across the experiment

Seven horses received antibiotics at least once from T1 to T5 (27 months apart) and showed clinically important physical findings during the experiment, including respiratory disorders, orthopedic injuries or systemic infections (Supplementary Table S3). In all of the cases, antibiotic treatment was administered at least 20 days prior to the sampling point (Supplementary Table S3), which ensured the return to the baseline gut microbiota before sampling^22^. Intestinal disorders such as colic, ventral edema and diarrhea were recorded in four individuals (2 control and 2 pasture) but none of them received antibiotics (Supplementary Fig. S2a and Supplementary Table S3). Around 50% of horses experienced orthopedic injuries during the study, *e.g.*, laminitis, injuries to the tendons situated at the palmar/plantar side of the equine distal limb and osteoarthritis. They were mainly treated with anti-inflammatory drugs (Supplementary Table S3). The risk of injuries was not associated with the group (trend test χ^2^ = 3.375, *p* = 0.066) and thus, horses kept alone in boxes presented the same level of injuries than horses kept in pasture with conspecifics.

### Effects of a temporary period at pasture on the fecal microbiota

The fecal microbial communities of all individuals distributed in the control or pasture group were tracked from T1 to T5. Stool samples were analyzed at baseline (from T1 to T3) and after the return to the individual boxes (T4 and T5). Each fecal sample was characterized via next-generation sequencing of V3–V4 hyper variable region of the 16S rRNA gene, allowing for a total of 16,536,376 high-quality sequences reads (mean per subject: 59,468 $\pm$ 38,030; range: 10,374–161,579). Reads were clustered into 7,628 chimera- and singleton-filtered Amplicon Sequence Variants (ASVs) at 99% sequence similarity (Supplementary Table S4).

**Figure 3:**
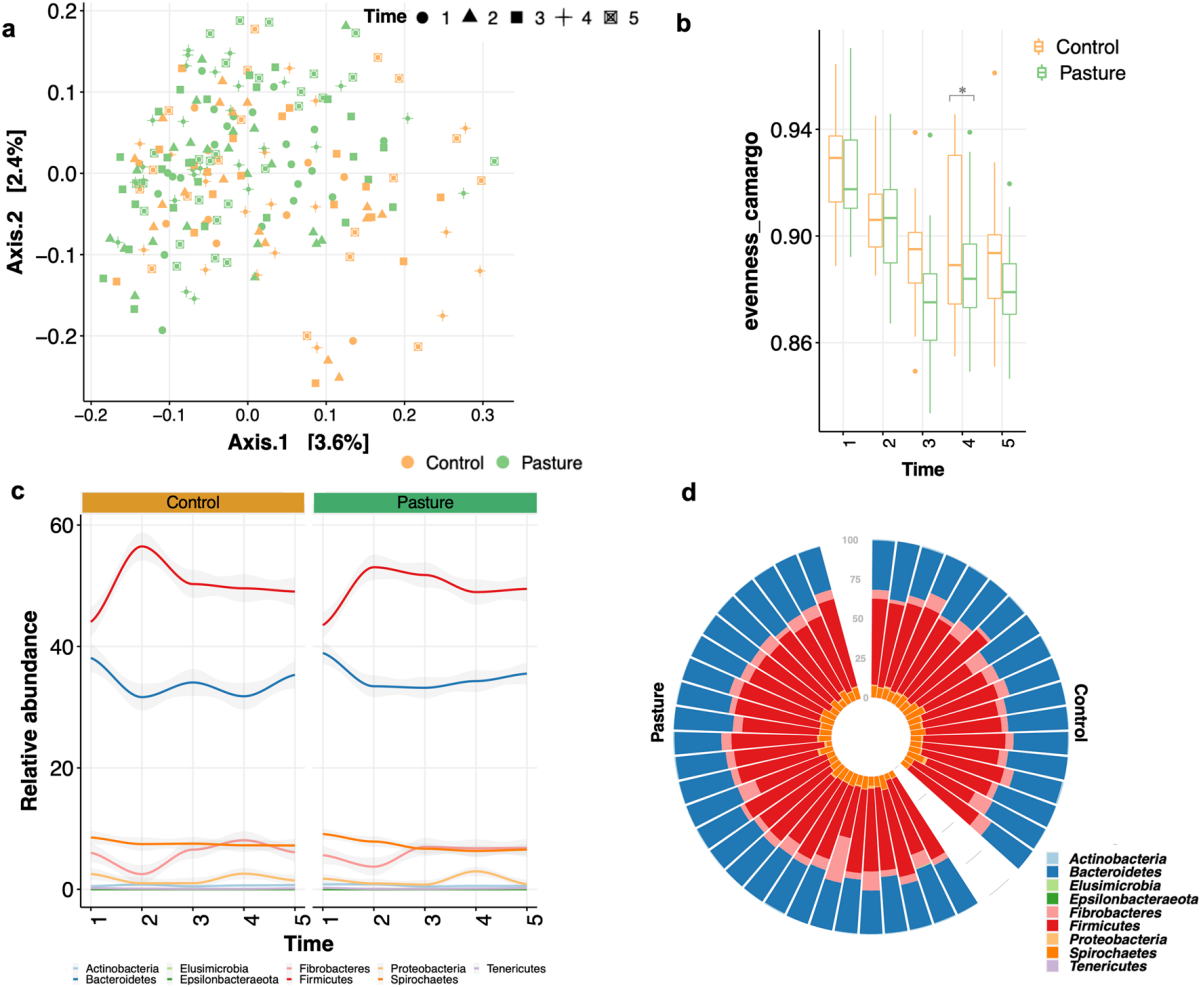
Description of the bacterial community diversity and the main phyla between groups. (**a**) Dissimilarities in fecal microbiota composition represented by the non‐metric multidimensional scaling (NMDS) ordination plot, with Bray–Curtis dissimilarity index calculated on un-scaled ASVs abundances. Samples are colored by group: pasture (green) and control (orange). The shape of dots indicates the longitudinal time point (from 1 to 5); (**b**) box plot representation of the evenness Camargo index using the rarefied ASVs table for each group and time points. The box plot features the median (center line) and interquartile range, and whiskers indicate 5th to 95th percentile; (**c**) Longitudinal evolution of the most abundant phyla of horse gut microbiota in the control and pasture groups. Graph shows relative abundance (y axis) of the most common bacterial phyla over time (x axis), averaged across control and pasture groups; (**d**) circular stacked bar plot of the main phyla for each individual in the cohort. Rare phyla such as Elusimicrobia, Verrucomicrobia and Synergistetes are not represented (mean relative abundance across groups < 0.01%). Individuals are stratified by groups. All plots were produced in R software https://www.r-project.org, using the version 4.0.2.

The non-multiple dimensional scaling (NMDS) based on weighted UniFrac distance or Bray–Curtis dissimilarity was performed to investigate differences in the baseline microbiota and to analyze the influence of a short period out to pasture on microbiota distribution at the ASVs and phylum levels. The ß-diversity of fecal microbial communities was similar when comparing bacterial communities longitudinally for control and pasture horses at any time point (Fig. 3a). The fact that individuals did not fall into distinct bins based on pasture or control group was also verified by permutational multivariate analysis of variance (PERMANOVA*, p* > 0.05), which tested the effects of group and time corrected by breed, sex, equine specialty and equine discipline on the variation of total dissimilarity between fecal microbiota. The patterns of variation in bacterial community structure were neither related to the level of competition, nor to training frequency, clinical disorders along the experiment and antibiotic administration (Supplementary Fig. S3a–d). Moreover, the fecal pH, which is a good proxy of perturbations in the intestinal microbiota due to gastrointestinal diseases^23–25^, antibiotics administration and the stress itself^26^ was also not associated with the microbiota community variation (Supplementary Fig. S2b).

Short exposure to pasture was not significantly associated with an increase or decrease in all common measures of α-diversity over the longitudinal time course (*p* > 0.05; SplinectomeR’s *permuspliner* function). Only, Camargo’s index of evenness, which is an excellent measure for community structure and it is unaffected by species richness, were significantly lower in pasture individuals compared to control horses after returning to the individual boxes (between T3 and T4 time points; *p* = 0.03, SplinectomeR’s *permuspliner* function; Fig. 3b), meaning that the community was dominated by one or a few species^27^.

**Figure 4:**
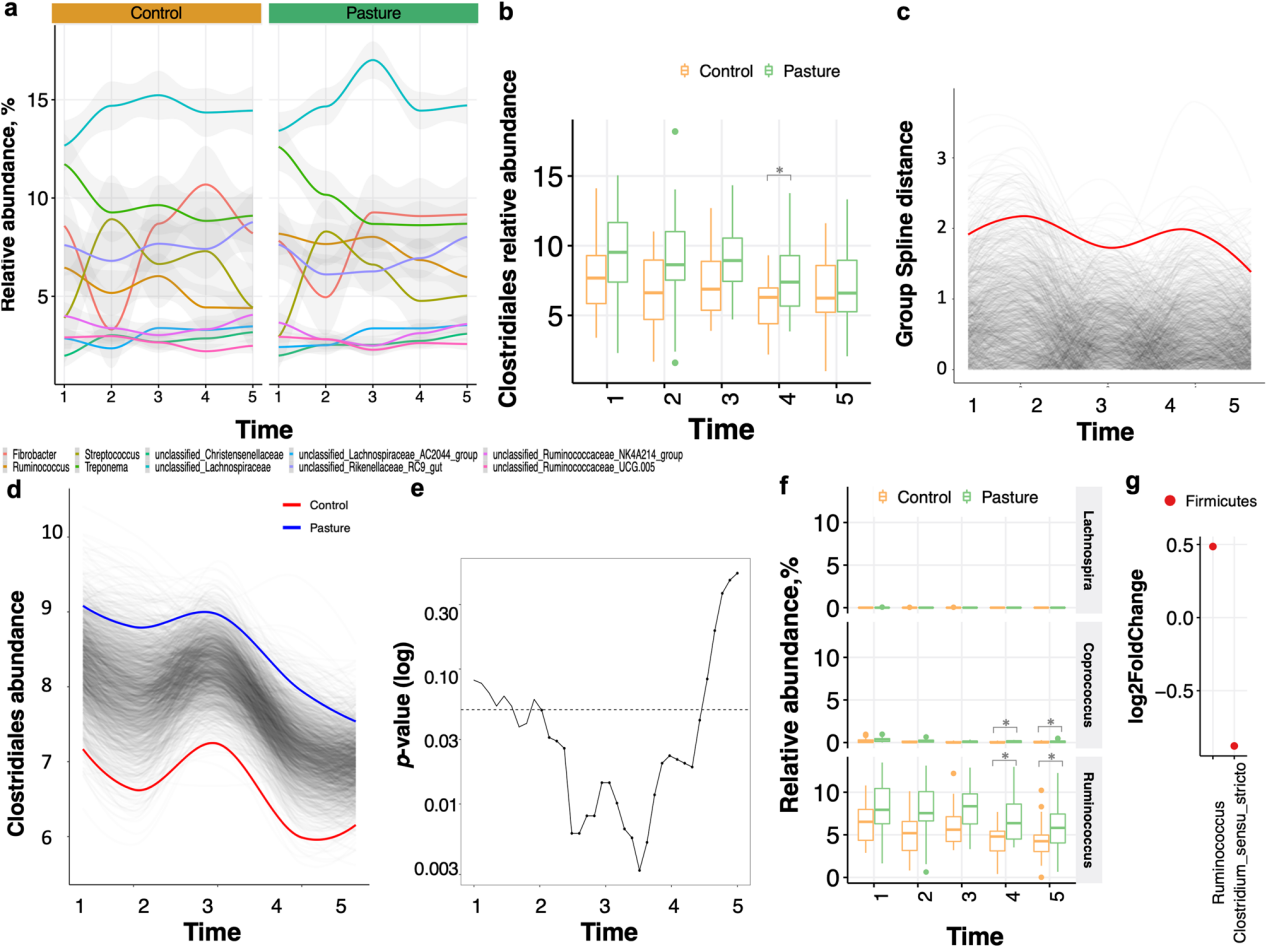
Longitudinal fecal microbiota composition differences between groups. (**a**) The longitudinal variation of the 10 most abundant genera in the horse fecal microbiota between groups. Graph shows relative abundance (y axis) of the most common bacterial genera over time (x axis), averaged across control and pasture group; (**b**) box plot representation of the relative abundance of Clostridiales between groups and across time. The box plots feature the median (center line) and interquartile range, and whiskers indicate 5th to 95th percentile; (**c**) permuted spline tests for statistical significance using the entire 27-month time series. Group distance plot for the Clostridiales comparison. The graph shows that permuted splines support a trend toward a greater true statistical difference after T3. The observed difference between the groups exceeded the random permuted distribution, which supports the statistically significant finding (*p* < 0.05); (**d**) Clostridiales abundance was different between pasture group (group spline in blue) and control horses (group spline in  red; 9,999 permutations,* p* < 0.05); (**e**) the plot output of the *sliding spliner* function shows a window of significance between the T3 and T4 time points. The *p*-value at each specified interval derived from the distribution of points from individuals’ smoothed splines. Dotted line indicates *p* = 0.05; (**f**) box plot representation of the relative abundance of the single taxa included within Clostridiales between groups and across time. The box plots feature the median (center line) and interquartile range, and whiskers indicate 5th to 95th percentile. Asterisk indicates *p* < 0.05; (**g**) dot plot representation of log-transformed fold change of genera that were significantly different between pasture and control across time using DESeq2. The log of fold changes superior to 0 indicate that genera were more abundant in pasture individuals than control individuals at any time of the experiment. By contrast, the log of fold changes inferior to 0 indicate that the genera abundances were lower in pasture relative to control individuals. Dots are colored by phyla. All plots were produced in R software https://www.r-project.org, using the version 4.0.2.

The microbiota composition between groups and their temporal dynamics were further analyzed by evaluating the specific phyla and genera propensity to variation between groups over time. The gut bacterial phyla did not differ in abundance between pasture and control group at any point in time during the time series (Fig. 3c,d). In both groups, Firmicutes (49.6 ± 6.23%) outranked Bacteroidetes (34.6 ± 5.12%), Spirochaetes (7.4 ± 2.41%) and Fibrobacteres (5.9 ± 3.78%) phyla (Fig. 3c,d). This trend was bolstered at the genus level, whose single relative abundance was similar between groups at any time point (Fig. 4a; supplementary Table S5). Notable, however, pasture exposed individuals presented an enrichment in butyrate-producing Clostridiales (specifically *Ruminococcus* and *Coprococcus*) compared to non-exposed individuals (*p* = 0.004, SplinectomeR’s *permuspliner* function; Fig. 4b–d), with a clear temporal pocket of significance between the T3 and T4 time points (Fig. 4e, SplinectomeR’s *sliding spliner* function). Accordingly, the relative abundance of *Ruminococcus* was significantly higher in pasture group compared to control group across time after corrections for multiple comparisons (adjusted *p* < 0.05 with DESeq2; Fig. 4f–g; supplementary Table S6). *Coprococcus* tended to be greater in pasture group relative to control group at T5 (adjusted *p* = 0.11 with DESeq2; supplementary Table S6).

### Association between the fecal microbiota and behavioral parameters

**Figure 5:**
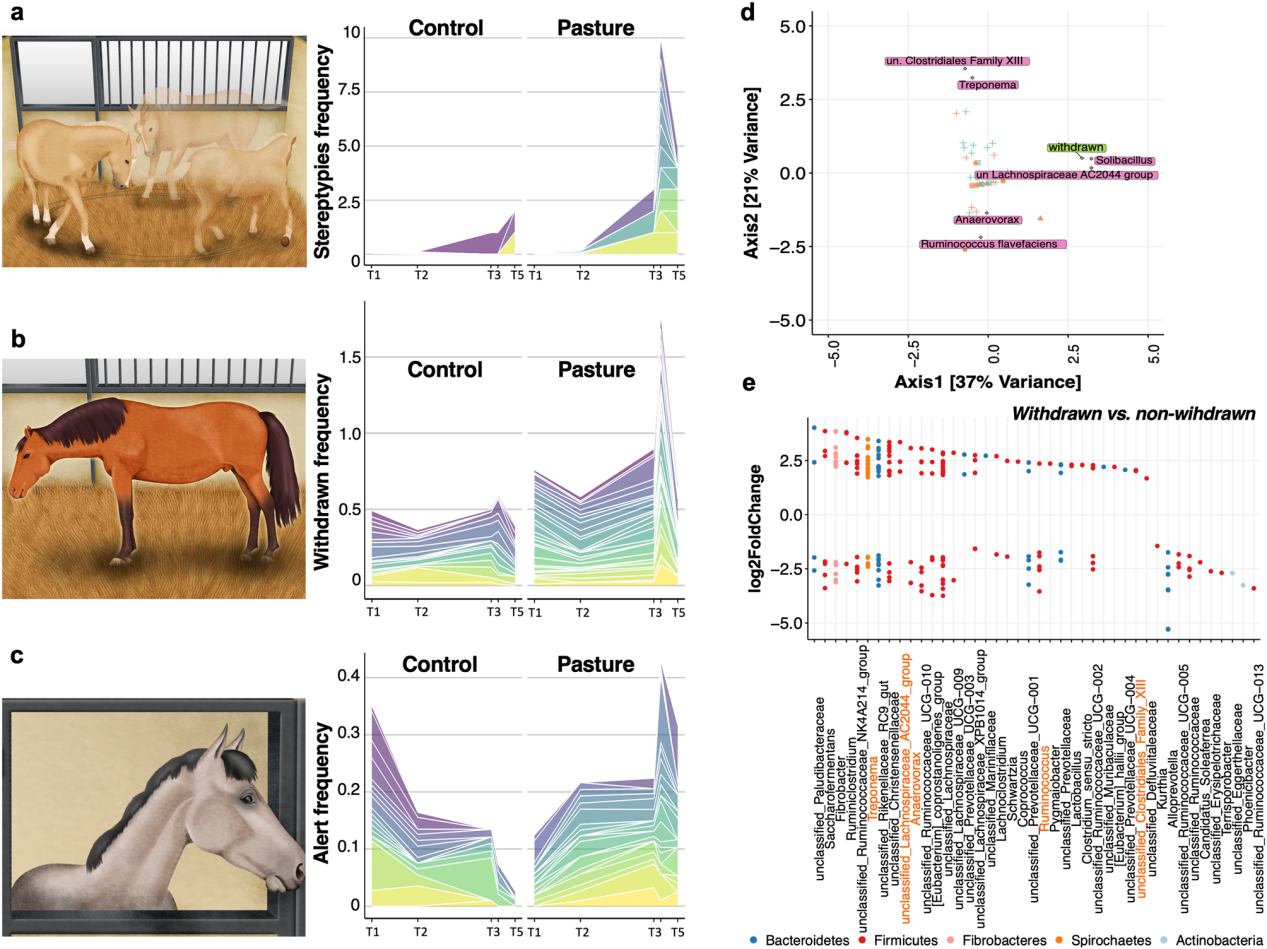
Association between the temporal dynamics of the fecal microbiota and behavioral parameters three months post-pasture. (**a**) Stacked area chart displaying the evolution of stereotypies frequency in control and pasture groups across time; (**b**) stacked area chart displaying the evolution of withdrawn frequency in control and pasture groups across time; (**c**) stacked area chart displaying the evolution of alert behavior frequency in control and pasture groups across time. In all cases, the measurements were done at baseline time (from T1 to T3), immediately at the returning in the box and three months later (T5). The frequency values of the individuals within each group are presented on top of each other ; (**d**) sparse canonical correspondence analysis (CCA) ordination plot of ASVs abundance table and results of the analysis of behavioral variables affecting bacterial divergence. Significant behavioral variables are indicted as rectangles, and ASVs associated to the correspondent significant variable are depicted in diamonds with text labels. If the annotation at the specie level of each significant ASVs were not found, the genera was specified; (**d**) dot plot representation of log-transformed fold change of ASVs that were significantly different between horses expressing withdrawn behavior and those non-expressing the withdrawn behavior. The log of fold changes superior to 0 indicate that ASVs were more abundant in the individuals expressing withdrawn behavior. By contrast, the log of fold changes inferior to 0 indicate that the ASVs abundances were lower in individuals expressing withdrawn behavior. Dots are grouped by genera and colored by phyla. All plots were produced in R software https://www.r-project.org, using the version 4.0.2. The drawings corresponding to the stereotypy and withdrawn behaviors in the panel (**a**) and (**b**), respectively, have been obtained from Mach et al.^8^ and used according to terms of a Creative Commons Attribution 4.0. License: http://creativecommons.org/licenses/by/4.0/. No changes were made. Written permission for publication of the drawing concerning to the alert behavior (panel **c**) in the figure has been given.

When returning to individual boxes (the first five days), a strong occurrence of stereotypies (χ^2^ (1) = 3.86, *p* < 0.05), of the withdrawn (W (1) = 267, *p* < 0.05) and the alert postures (W (1) = 232.5, *p* < 0.001) was observed in pasture individuals compared to control horses (Fig. 5a–c), as reported elsewhere^19^. Furthermore, the pasture horses tended to express more alert postures than the control group up to 3 months after the return to the individual boxes (T5; W(1) = 160.5, *p* = 0.07; Fig. 5c). Therefore, we then sought to determine whether the increase occurrence of behaviors indicating deleterious effects on welfare significantly associated with the gut microbiota profiles. Data were analyzed at T5 (3 months after the return to the individual boxes) because simultaneously measurements of behavior indicators and fecal microbiota were available.

While the two-dimensional NMDS solution presented a moderate stress value of 0.15 (non-metric fit, *r*^2^ = 0.98; linear fit, *r*^2^ = 0.85), and none of the fitted behavior indicators correlated with the NMDS ordination of the microbiota structure (*envfit*; *r*^2^ < 0.03, *p* > 0.05), the constrained correspondence analysis (CCA) model built on the ordination of microbiota structure identified aggressiveness towards humans (*cca; r*^2^ = 0.98, *p* < 0.001) and the withdrawn posture (*r*^2^ = 0.92, *p* < 0.001) as explanations for the microbiota profiles at T5. The alert posture tended also to affect the ordination structure (*r*^2^ = 0.101, *p* = 0.11). Similarly, the PERMANOVA test demonstrated that the behaviors most strongly associated with community variation were withdrawn behavior (*p*_adj_ < 0.05) and the alert behavior (*p*_adj_ < 0.05). Additionally, the forward selection procedure based on the redundancy analysis (RDA) of microbiota identified withdrawn behavior as explanation for the microbiota profile (*forward.sel*; *r*^2^ = 0.04, *p* = 0.038).

To obtain a finer resolution of the interactions between the gut microbiota and behavioral dysregulation at T5, we employed the sparse canonical correspondence analysis (sCCA). For these taxon-covariate relationships, we found numerous associations involving butyrate-producing bacteria as well as other taxa of interest, including *Solibacillus,* members of the yet not classified *Lachnospiraceae* AC2044 group family*, Treponema* and bacteria from the Clostridiales family XIII, *Anaerovorax* genus and *Ruminococcus flavefaciens* (Fig. 5d). The robustness of these identified links was further studied through the DESeq2 differential ASVs abundance comparison method, which led to similar observations. For example, horses expressing withdrawn posture harbored a higher abundance of bacteria from the Clostridiales family XIII, members of the yet not classified *Lachnospiraceae* family*, Lactobacillus* and *Clostridium sensu stricto*, whilst horses not expressing withdrawn postures harbored significantly higher abundances of ASVs pertaining to *Kurthia, Alloprevotella* and members of the unclassified *Ruminococcaceae* UCG 005 specie (DESeq2, adjusted *p* < 0.05; Fig. 5e, supplementary Table S7). Intriguingly, ASVs within *Ruminococcus, Anaerovorax, Treponema* and other genera depicted in Fig. 5 were found to exhibit opposing abundance trends (Supplementary Table S7). Yet, *R. flavefaciens* were consistently less abundant in individuals expressing withdrawn posture compared to non-expressors (Supplementary Table S7).

## Discussion

This is the first study to assess if a temporary holiday on pasture modifies the gut microbiota and the occurrence of behaviors related to a compromised welfare state in elite horses.

The β-diversity of the gut microbiota was similar between groups and time series and was not related to equitation factors, clinical treatments and fecal pH. Whether the beneficial effect of the pasture environment induced short-term differences in the gut microbiota and host physiology and reverted immediately upon the return to the box remains to be elucidated. It is conceivable that the gut microbiota underwent a fast restoration of the baseline ecosystem following the time out to pasture, leading to a strong resilience of the communities. This notion of stability, however, was only considered from the standpoint of composition and functional changes following the pasture perturbation cannot be discarded.

Based upon individual taxa analysis, the temporary release to pasture increased the abundances of butyrate-producing Clostridiales (specifically *Ruminococcus* and *Coprococcus*) that lasted 1-month after the return to individual boxes, evoking beneficial effects on the immune and metabolic homeostasis^28,29^, as well as the host’s mental health^30^. Given the scarcity of green material in the pastures, the enrichment of butyrate-producing Clostridiales were unlikely due to differences in herbage intake. *Coprococcus* spp. are known to produce butyrate whereas *Ruminococcus* spp. consume hydrogen and mainly produce acetate via the acetyl-CoA pathway^31^. The functional consequences of such changes are not clear at this stage, but it is worth noting that in humans *Coprococcus* has been positively associated with quality of life indicators^17^. Moreover, in social-disruption-stressed mice the abundance of *Coprococcus*^32^ was decreased. Therefore, the beneficial effects of the pasture environment on the expression of natural behaviors could probably contribute to the increase of *Coprococcus*, which could be regarded as a potential psychobiotic^17^, a live organism that, when ingested in adequate amounts confers health benefits in patients suffering from emotional stress. Repeated exposures to pasture with conspecifics may be required to ensure a bloom of butyrate-producing bacteria such as *Ruminococcus* and *Coprococcus*, which in turn, may drive long lasting positive immune, metabolic and psychological changes in sport horses.

**Figure 6:**
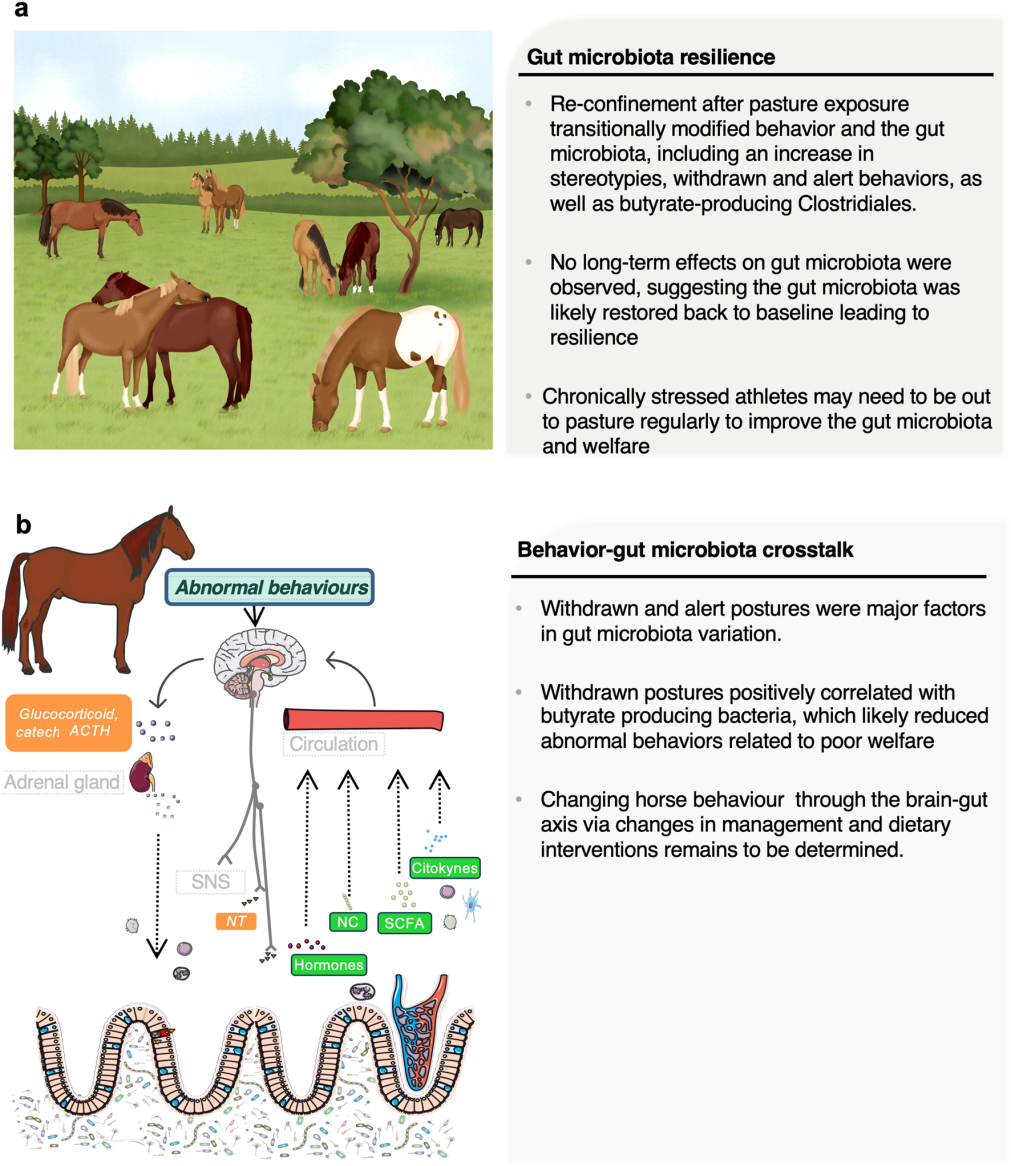
Main results and future hypothesis. (**a**) Key results obtained after the release of horse athletes in the pasture for a short-term period; (**b**) behaviors pointing to poor welfare as a modulator of gut microbial ecology. Abnormal behaviors might cause stress, thereby inducing the secretion of adrenocorticotropic hormone (ACTH), which in turn acts on the inner adrenal cortex to initiate the synthesis and release of glucocorticoid and catecholamines hormones, or by inducing the release of sympathetic neurotransmitters (epinephrine and norepinephrine). Additionally, the gastrointestinal tract responds to stress by releasing neuroactive molecules such as Gamma-amino butyric acid (GABA), neuropeptide Y and dopamine that have been purported to be involved in the gastrointestinal disturbances, anxiety, depression, reduced food intake and less stress coping (reviewed by Clark and Mach^74^). These stress molecules might influence bacterial gene expression or signaling between bacteria, and this might change the microbial composition and function of the microbiota. The putative mechanisms by which the gut microbiota interacts with the brain and influences behavior in athletes include bacterial subproducts (SCFA, H_2_S, indolic compounds, secondary bile acids), the synthesis or stimulation of endogenous neuroactive substances like (GABA, dopamine and serotonin) that gain access to the brain via the bloodstream, via cytokine release from mucosal immune cells, or via afferent neural pathways, including the vagus nerve. However, the extent to which the gut microbiota-gut-brain axis, and modulation of the gut microbiota through this axis influences behavior in athletes remains to be determined. *ACTH* adrenocorticotropic hormone, *NT* neurotransmitters, *NC* neuroactive compounds, *SCFA* short chain fatty acids. Written permission for publication of the drawing in the (**a**) panel has been given. In the (**b**) panel, the picture of the horse was downloaded from https://smart.servier.com/wp-content/uploads/2016/10/Animals.ppt. The image of the brain was download from https://smart.servier.com/category/anatomy-and-the-human-body/nervous-system/brain/. The image of the gut intestinal cells was downloaded from https://smart.servier.com/smart_image/intestinal-villi/. The kidney image was download from https://smart.servier.com/smart_image/kidney-2/. The immune cell representation and the blood capillary were obtained from https://smart.servier.com/category/anatomy-and-the-human-body/cardiovascular-system/blood/ . In all cases, no changes were made. Servier Medical Art by Servier is licensed under a Creative Commons Attribution 3.0 Unported License.

It is now clear that mental illnesses, such as anxiety disorders, are linked to gut dysbiosis^14,15^. In agreement, we identified the withdrawn and alert postures in elite horses as the major microbiome covariates, and thus, partially accounting for the variation in the taxonomic composition of the gut microbiota. We therefore hypothesize that abnormal behaviors caused by poor welfare promoted stress, and thereby, stimulated the sympatho-adrenomedullary (SAM) and hypothalamus–pituitary–adrenal (HPA) axes, resulting in the release of catecholamines and glucocorticoids into the circulatory system which could alter the gut microbiota (Fig. 6). In line with this, the frequency of withdrawn behavior was positively associated with the prevalence of bacteria from *Lachnospiraceae* AC2044 group and Clostridiales family XIII, which include butyrate-producing bacteria that exert important pleiotropic functions in the intestinal tract and beyond^33,34^. As suggested in our previous study^8^, a synergistic effect could exist between the host and butyrate-producing bacteria to reverse or counteract the potentially negative effects of abnormal behaviors. Likewise, previous studies in chronically stressed mice have shown that butyrate levels strongly associated with improvement in anxiety- and depression-like behavior tests^35^ and that butyrate can reverse the enduring effects of psychosocial stress^36^. Although we cannot definitely prove a causal link between changes in abnormal behaviors and the gut microbiota in horses at this juncture, concomitantly acting upon gut microbiota might be a plausible mechanism for how management interventions can break the vicious cycle of depression and restore welfare. Despite these findings, our study showed that withdrawn behavior was negatively associated with the abundance of *Ruminococcus flavefaciens*. This observation defies our expectation given that prior animal studies have shown *R. flavefaciens* plays an important role in sustaining depression^37^ and the importance of decreasing *R. flavefaciens* for antidepressants to achieve their therapeutic effect. It is possible that *R. flavefaciens,* the predominant cellulolytic bacterial specie of the equine cecum^38^, carries out other functions in horses than inducing changes in cortical gene expression while down-regulating genes involved in neuronal plasticity^37^.

In summary, the global microbial gut community exhibited resilience after pasture exposure, yet functionally redundant butyrate-producing bacteria with positive health outcomes, namely *Ruminococcus* and *Coprococcus,* were increased following a period out to pasture. Moreover, withdrawn posture was associated with higher abundance of butyrate-producing bacteria, supporting the hypotheses of an influence of the depressive-like behavior on gut microbiota in horse via the gut-brain axis. Future studies investigating the crosstalk between gut microbiota and behavior on multiple timescales will open the possibility of microbiota-targeted strategies to contrast welfare decline in sport horses.

## Methods

### Animals and treatment groups

The present study included a total of 45 elite sport horses that had lived in individual boxes since they were three years old and cared for under similar conditions. The animal cohort and criteria of inclusion have been previously described by Mach et al.^8^ and Ruet et al.^19^. Horses were recruited from a cohort of 376 individuals housed at the French National Riding School, in Saumur (France). Out of 376 individuals, 185 were included in the study by Mach et al.^8^ and 60 were used for the study by Ruet et al.^19^. The 45 individuals enrolled in the present study were shared by both, Mach et al.^8^ and Ruet et al.^19^.

As described by Ruet et al.^19^, the selected horses were randomly split into two groups: 18 horses (11 geldings and 7 mares) aged 9.16 ± 3.18 years (mean ± SD) were kept in individual boxes (control group) whereas 27 horses (17 geldings and 10 mares) aged 10.29 ± 2.50 years were moved to pasture (pasture group). Horses in the control group were permanently housed in individual boxes of 9 m^2^, which prevented individuals from performing natural behaviors such as grazing and moving freely, unless they were exercised. All horses had visual contact with conspecifics, but none could engage in social interactions. Because jump racing, dressage and endurance competitions in France generally take place from early summer to autumn months, control horses were continuously trained at high intensity for sport purposes six days a week during the study (Supplementary Table S1).

Horses in the pasture group were sent to poor pasture for 41.7 ± 3.03 days. Pastures were located 5 ± 1.5 km from the riding school^19^. The average surface area of the pastures was much larger than the minimum recommended, ensuring a low level of aggression among horses (0.03 hectares per horse^39^). All pastures were equipped with one or two man-made shelters. The senescence of green material in the pastures was scant with no herbage growth because of high heat temperatures (26 ± 4.15 °C) and lack of rainfall (0.5 ± 0.73 mm/day) during August 2018. Following the turned-out on to the poor pasture, horses were trained at high intensity for sport purposes six days out of seven (Supplementary Table S1) and they were permanently housed in individual boxes of 9 m^2^ (as for control group).

The number of subjects in the control group was smaller than in the pasture group based on the ‘Anna Karenina principle’^40^. The peculiar name of this principle from the novel by Leo Tolstoy, which starts with the sentence: ‘All happy families look alike, each unhappy family is unhappy in its own way’. We assumed that the gut microbiota composition of individuals facing a temporary perturbation (pasture with conspecifics) could vary more than that of individuals not exposed to environmental challenges, that is, permanently housed in individual boxes.

For successful participation in the experiment, we required the following criteria: (1) written informed consent from the owners; (2) access to behavior and microbiota profiling data across the study; (3) horses had been kept in the individual boxes since they were three years old; (4) the animals expressed one or more behavioral indicators of a compromised welfare state before going out to pasture; (5) data registration about concentrate composition and intake throughout the experiment.

Exclusion criteria included antibiotic treatments and immune modulating agents within 20 days prior to the sampling point or anthelmintic medication within 60 days before the sampling point. Food additives, prebiotics, probiotics or symbiotics intake that could affect gut microbiota composition were not allowed during the study.

### Equitation variables

For each individual, we recorded equitation factors such as the discipline (eventing, dressage, show-jumping or vaulting), the specialty, the level of performance and the training frequency (*e.g.*, hours being ridden, trained on the lunge or exercised with an automatic walker per week, as well as their levels of performance). The equine specialties recorded were gala, Cadre Noir, competition and formation. As described elsewhere^8^, Gala horses were high-level performers ascribed to high level of physical and mental engagement (with lots of transport and public shows). The Cadre Noir horses were those trained to become a regular high-level performer, while competition horses were medium-level performers and were subjected to punctual and high/medium level of physical and mental engagement. Lastly, formation horses were those used for training candidate riders in the riding arena of the French National Riding School. Concerning the level of competition, horses were engaged in a low level of performance (*amateur* category), trained for high-level competitions (professional category) or have achieved the highest level of performance in international competitions or an equivalent level of training (expert category; Supplementary Table S1).

### Feeding variables

Detailed macronutrient profiles were calculated between groups at each time point (Supplementary Table S2). Dietary intake was recorded from each individual each day of the study and averaged by week or fortnight to reflect habitual dietary intakes around the sampled time points. All of the individuals (pasture and control) were fed with the same type of commercial concentrate pellets, which were designed for performance horses. The commercial concentrate pellets (Flaked Royal Horse, Saint-Nolff, France) consisted of flaked barley, alfalfa, flaked corn, soybean teguments, wheat straw, sugar beet pulp, molasses, extruded soybeans, rapeseed oil, flax oil, salt, carbonate Ca and a mineral and vitamin mix. The mineral and vitamin mix contained Ca (28.5%), P (1.6%), Na (5.6%), vitamin A (10,000 IU), vitamin D_3_ (1500 IU), vitamin E (200 IU), vitamin B_1_ (12 mg/kg), cobalt carbonate (0.3 mg/kg), cupric sulfate (25 mg/kg), calcium iodate (0.4 mg/kg), iron sulfate (90 mg/kg), manganese sulfate (50 mg/kg), sodium selenite (0.30 mg/kg), and zinc sulfate (80 mg/kg). The average daily intakes of macronutrients listed on the commercial concentrate pellets were calculated according to the detailed descriptions of the respondents^13^. The word fiber within the macronutrients list refers to structural fiber, *e.g.,* which has not been chopped or ground down through processing.

The aforementioned commercial concentrate was the only source of concentrate offered to the 45 horses. In control horses, concentrate was fed individually in stalls divided into three daily meals. For the individuals sent out to pasture, concentrated feed was offered and controlled individually by a caretaker who also monitored the animals every day to ensure that they were not sick or injured.

Commercial concentrate was offered in amounts dependent of the horses’ body weight and training intensity. For most of individual, the quantity of concentrate was kept equal throughout the training period. However, the concentrate regime of several horses was reduced from T4 onwards because of high body condition and excess weight (Supplementary Table S2). Moreover, the amount of concentrate offered in horses turned out to pasture was reduced by ~ 500 g/day (Supplementary Table S2). Abrupt reductions of the amounts of concentrate offered in horses turnedout to pasture were discarded to avoid digestive and metabolic disorders that could impair the performance of horses.

Daily concentrate intake was calculated as the difference between the amount offered and the amount which remained un-eaten.

All horses received 9.2 ± 0.15 kg of hay per day, regardless of their needs.

The total macronutrients intake was then estimated by multiplying the frequency of the ingredient consumption and its nutrient content^13^ (Supplementary Table S2). The UFC (“Unités Fourragères Cheval”) or horse feed units value of a feed is equal to the ratio between the net energy content of feed and the net energy content of barley^41^, whereas the net energy and digestible protein is expressed as “Matières Azotées Digestibles Cheval”(MADC) per kg of dry matter.

Water was available ad libitum.

### Clinical variables

Veterinary surgeons from the ridding school provided the information that they collected on the presentation of clinical abnormalities (including colic) or fever in any horse along the investigation period (Supplementary Table S3). The treatment and diagnosis are detailed in the supplementary Table S3.

### Longitudinal measurements of behavior for each individual

Behavior indicators were studied during five different periods along the study, that is, before pasture (T1, T2, T3),  five days after returning to the box and three months after the return in the box (T5). For each time period, the horses were observed over five consecutive days using the scan sampling method. The details of the total number of scans recorded per period and per group, as well as the methodology applied are described in the studies performed by Ruet et al*.*^3,19^. Briefly, four independent behavioral indicators reflecting compromised welfare state were recorded during the experiment: stereotypies, aggressive behaviors towards humans, the unresponsiveness to the environment and the alert posture. Stereotypies included oral behaviors such as crib biting or locomotion behaviors such as weaving, nodding and head bobbing^3,19^. Aggressiveness towards humans included a continuum of behaviors from ears pinned backward to biting, or kicking^3,19^. Unresponsiveness to the environment was identified through a particular posture called the withdrawn posture in which horses stood with the neck horizontal at the same level as back, with fixed stare^3,19^. Finally, an internal state of hypervigilance was identified through the alert posture, described as elevated neck, ears pricked forward and looking intensely at the environment^3,19^. The percentage of scans recorded for each behavioral indicator per period was calculated from the total number of observations per horse ([number of scans in which the horse expressed the indicator / total number of scans] × 100)^19^. As described by Ruet et al.^19^, the percentage of scans of aggressive behaviors towards humans when horses were in individual boxes, and that of the withdrawn and alert postures were analyzed as continuous variables. However, stereotypies were treated as the proportion of horses expressing this indicator per period because of the high number of null values among the simple^19^.

All of the horses were used to road transportation. However, it can be not discarded that direction, duration of the transport and how horse were loaded into the truck affect the stress response and the expression of the aforementioned behaviors^13,42,43^.

### Fecal microbiota across time for each individual

The fecal microbial profiling of the 45 horses were tracked at five different time points that spanned from October 2016 to December 2018 (27 months apart); that is, October 25^th^, 2016 (T1), June 19^th^, 2017 (T2), July 3^rd^, 2018 (T3), September 17^th^, 2018 (T4) and December 17^th^, 2018 (T5).

The first two time points corresponded to those already reported in our previous studies^8,44^. They were included in the current study to enhance our understanding of the baseline gut microbiota and to better determine the microbial features that when disrupted by the short-term exposure at pasture could be modified and eventually associated with host behaviors. The T3, T4 and T5 time points corresponded to pre-pasture, and one and three months after the return to the box, respectively. Since diet is a major factor that influences and shapes the gut microbiome in horses^11,45–51^, we decided to profile the microbiota at least one month after the return to the box to avoid a confounding effect of pasture intake in the pasture group. This decision relied on several studies^48,49,52–57^. Briefly, most studies assessing the impact of an abrupt change of diet composition on the horse hindgut microbiota reported significant temporary shifts during the first hours^48,49,52–55^, with no longer differences between diets at seven days^56^. However, the total anaerobes, lactobacilli, streptococci and lactate utilizers counts within the subsequent three weeks following the change of silages with different levels of crude protein^57^.

Fecal samples were collected from the rectum at each time point as described in our previous studies^8,44,58,59^. Fecal samples were collected because they provide a reasonable profile of the microbiota luminal composition of the equine large intestine^60^. Precisely, fecal microbiota are highly similar to the microbiota of the dorsal colon but include many of the microbial taxa present in the ventral colon and cecum^61^, in which the bulk of fiber fermentation takes place. Approximately 10 g of feces were collected from the center of several fecal balls, avoiding collection of fecal material that was touching the veterinarian globes. Fecal aliquots for microbiota analysis were immediately snap-frozen in liquid nitrogen and stored at − 80 °C until DNA extraction, whereas fecal aliquots to measure the fecal pH were immediately sent to the laboratory. The fecal pH was determined after 10% fecal suspension (wt/vol) in saline solution (0.15 M NaCl solution).

### Ethical statement

The local animal care and use committee reviewed and approved the study protocol (CEEA Val de Loire; reference: 2019012211274697.V4-18939, dated March 29, 2019). All protocols were conducted in accordance with EEC regulation (no 2010/63/UE) governing the care and use of laboratory animals, which has been effective in France since the 1^st^ of January 2013. In all cases, owners and riders provided their informed consent prior to the start of study procedures with the animals. Additionally, for the evaluation of horse aggressiveness towards humans, all methods were carried out in accordance with the Declaration of Helsinki. All experimental protocols were approved by the CEEA Val de Loire (reference: 2019012211274697.V4-18939) and informed consent was obtained from all subjects.

### Microorganisms DNA extraction from fecal samples

Total DNA was extracted from aliquots of frozen fecal samples (200 mg), using E.Z.N.A. Stool DNA Kit (Omega Bio-Tek, Norcross, Georgia, USA). The DNA extraction protocol was carried out according to the manufacturer’s instructions (Omega Bio-Tek, Norcross, Georgia, USA). DNA was then quantified using the Qubit dsDNA HS assay kit (Thermo Fisher Scientific, Waltham, MA, USA).

### V3–V4 16S rRNA gene amplification

The V3-V4 hyper-variable regions of the 16S rRNA gene were amplified with two rounds of PCR as previously described in our lab^8,44,58,59^. The concentration of the purified amplicons was measured using Nanodrop 8000 spectrophotometer (Thermo Fisher Scientific, Waltham, MA, USA) and the quality of a set of amplicons was checked using DNA 7500 chips onto a Bioanalyzer 2100 (Agilent Technologies, Santa Clara, CA, USA). All libraries were pooled at an equimolar concentration in order to generate equivalent number of raw reads within each library. The final pool had a diluted concentration of 5 nM to 20 nM and was used for sequencing. Amplicon libraries were mixed with 15% PhiX control according to the Illumina’s protocol. For this study, one-sequencing run was performed using MiSeq reagent kit v2 (2 × 250 output; Illumina, San Diego, CA, USA) for T1 and T2. Another sequencing run was performed using MiSeq reagent kit v3 (2 × 250 output; Illumina, San Diego, CA, USA) for T3, T4 and T5 time points, respectively.

### V3–V4 16S rRNA gene sequencing and data pre-processing

The Divisive Amplicon Denoising Algorithm (DADA) was implemented with the DADA2 plug-in for QIIME 2 (version 2019.10) to perform quality filtering and chimera removal and to construct a feature table consisting of read abundance per ASV by sample^62^. DADA2 models the amplicon sequencing error in order to identify unique ASV and to infer sample composition more accurately than traditional Operational Taxonomic Unit (OTU) picking methods that identify representative sequences from clusters of sequences based on a % similarity cut-off^62^. The output of DADA2 was an abundance table, in which each unique sequence was characterized by its abundance in each sample. Taxonomic assignments were given to ASVs by importing SILVA 16S representative sequences and consensus taxonomy (release 132, 99% of identity) to QIIME 2 and classifying representative ASVs using the naive Bayes classifier plug-in^63^. The feature table, taxonomy and phylogenetic tree were then exported from QIIME 2 to the R statistical environment and combined into a phyloseq object^64^. Prevalence filtering was applied to remove ASVs with less than 1% prevalence and in less than three individuals, decreasing the possibility of data artifacts affecting the analysis^62^. To reduce the effects of uncertainty in ASV taxonomic classification, we conducted most of our analysis at the microbial genus level.

The phyloseq^65^, vegan^66^ and microbiome R packages were used for the detailed downstream analysis. The minimum sampling depth in our data set was 10,423 reads per sample. Before the estimation of diversity indexes, samples were rarefied at 10,000 reads of depth, to allow an equal depth using the *rarefy_even_depth* function in the phyloseq R package, which is implemented as an *ad hoc* means to normalize microbiome counts that have resulted from libraries of widely differing size. The minimal sequencing depth of 10,000 was sufficient for accurately profiling bacterial composition, as predicted by the calculation of the rarefaction curve for observed richness and Shannon index (which accounts for both abundance and evenness).

ASV counts per sample and ASV taxonomical assignments are available in supplementary Table S4. Data was aggregated at genus, family, order, class and phyla levels throughout the taxonomic-agglomeration method in phyloseq R package, which merges taxa of the same taxonomic category for a user-specific taxonomic level.

### Statistical analysis

#### Differences in total DMI, concentrate intake and daily macronutrient ingestion between groups

The total DMI, concentrate intake and macronutrient intakes were statistically compared between pasture and control groups at each time point by using normal linear mixed models (lme4 R package)^67^ with group and time fitted as fixed effects and horse as random effect to account for inter-horse variation. Conditional (*R*^2^*c*) and marginal (*R*^2^*m*) coefficients of determination were calculated using the function *lmer* in the R package lme4. The marginal *R*^2^*m* represents the variance explained by fixed effects in the model, whereas the conditional *R*^2^*c* accounts for the variance explained by fixed effects and random effects combined. The ICC was calculated by dividing the between-group-variance (random intercept variance) by the total variance (*e.g.*, sum of between-group-variance and within-group; residual variance). In our model, the ICC quantifies the correlation between measurements on the same individual (intra-horse variability). Higher the value, higher the correlation between time points.

#### Behavioral comparisons between pasture and control groups

Wilcoxon rank-sum tests with continuity correction (*wilcox.test* function in the stats R package) were used to compare the percentages of scans of aggressive behavior towards humans, the withdrawn and alert postures between pasture and control horses per period. The proportion of horses with presence or absence of stereotypies was compared between the two groups using χ^2^ tests of homogeneity (*chisq.test* function in the stats R package). All statistical analyses were performed using R software with a significance level of *p* < 0.05.

#### Orthopedic injuries prevalence between pasture and control groups

The prevalence of orthopedic injuries in horses was compared between the two groups using χ^2^ tests of homogeneity (*chisq.test* function in the stats R package). All statistical analyses were performed using R software with a significance level of *p* < 0.05.

#### α-diversity indices of the fecal microbiota between groups and its temporal dynamics

The α-diversity indices were calculated using the phyloseq and microbiome R packages from ASV and relative genera abundance tables, as described in our previous studies^8,44^. Shortly, the microbiome R package enabled to study global indicators of the gut ecosystem state, including measures of evenness, dominance, divergences and abundance.

The microbial α-diversity metrics between the sampling points and groups were compared using the splinectomeR tool^68^, which contains three primary functions to test specific hypothesis about longitudinal trends. Specifically, the *permuspliner* function enables to test whether two groups of samples follow more different trajectories over time than would be expected by random chance^68^. It compares two groups over time without collapsing the timepoints to a single averaged point. As a complement to the *permuspliner* test, the *sliding spliner* function allows the user to test the data series at defined intervals and ask whether the two groups of interest are significantly different at any point in time during the time series^68^. Lastly, the *trendyspliner* function tests whether a set of responses in one group changes in a non-zero direction over the time series (or other continuous independent axis)^68^. A spline is fit to the data and interpolated across the number of set intervals. Non-zero change is measured as the area between the group spline and a linear baseline that is established from the start point of the group spline^68^.

#### β-diversity of fecal microbiota between groups and its temporal dynamics

To estimate β-diversity, un-weighted and weighted UniFrac distances, as well as Bray–Curtis dissimilarity, were calculated from the ASV, phyla and the genera abundance tables. The β-diversity was visualized using the NMDS through the phyloseq and vegan R packages.

The among-group and time differences in the gut microbiota composition were tested through the PERMANOVA. Specifically, we tested the effects of group and time corrected by breed, sex, equine specialty and equine discipline on the variation of total dissimilarity between fecal microbiota. Although breed exerts limited effects on the equine fecal microbiota^44^, it is recommended to account for breed variation as a potential confounding factor in studies linking microbiota differences to host phenotypes in horses. Equine specialty and discipline have been associated with different degrees of physical and mental stress during training and show events, which altered intestinal microbiota composition^8^; therefore, their inclusion as cofactors in the model was highly recommended. The significance of the effect of time was assessed in an *F*-test based on the sequential sum of squares estimated from a 10,000 permutations procedure. The significance threshold was chosen at adjusted *p* < 0.05. The effect of level of competition, training frequency, type of clinical disorders, antibiotic administration and fecal pH on the ASV level community ordination was tested using the *envfit* function in the vegan R package (10,000 permutations, Benjamini–Hochberg multiple test correction *p* < 0.05). Fecal pH was categorized based on the calculation of interquartile ranges and standard deviations.

#### The contribution of each genus to the ecosystem temporal dynamics through groups

By employing the SplinectomeR tool functions^68^, we explored overall changes in genera abundances and compared longitudinal trends between pasture and control groups. Additionally, the DESeq2^69^ R package was used to test for differential genera abundances between groups across the experiment. DESeq2 assumes that counts can be modeled as a negative binomial distribution with a mean parameter, allowing for size factors and a dispersion parameter. Next to the group and time factors, the horse dependency was included in the generalized linear model. The *p*-values were adjusted for multiple testing using the Benjamini and Hochberg procedure. DESeq2 comparisons were run with the parameters fitType = “parametric” and sfType = “poscounts”.

#### Associations between gut microbiota and behavioral indicators

These associations were run at three months post-pasture; T5. We tested for associations between the ASVs and genus-level community composition and host behavioral indicators using the NMDS based on Bray–Curtis dissimilarity ordination method followed by the *envfit* function^70^ in the vegan R package, with 10,000 permutations and Benjamini and Hochberg multiple testing correction. An adjusted *p* < 0.05 was considered as significant. This function performed a multivariate analysis of variance (MANOVA) and linear correlations for categorical and continuous variables, respectively, and enabled the selection of combined covariates with strongest correlation to the gut microbiota variation. Moreover, we used the CCA^71^ to determine the variance in microbiota composition that covaried with the horse behavioral dysregulation variables. We assessed the significance of associations between the selected behavioral covariates and the microbiota using the PERMANOVA analysis (*anova.cca* function) on the resulting CCA ordination^71^. We also used a forward selection with double stopping criteria (*p* < 0.05 and adjusted *r*^2^ less than that found with all vectors), as described by Blanchet, Legendre and Borcard^72^. This was implemented using the functions *RsquareAdj* and *forward.sel* from the R package adespatial. The cumulative contribution of behavioral variables was determined by forward model selection on a distance‐based redundancy analysis (dbRDA) with the *ordiR2step* function in vegan^71^.

The above-mentioned methods can test the relationships between the covariates of interest and the overall composition of the gut microbiota, but they may miss significant interactions between host covariates and individual taxa (*e.g.,* ASVs or genera). To determine if such relationships existed in our data, we conducted the sparse CCA, a method well-suited to both exploratory comparisons between samples and the identification of features with interesting variation (PMA R package^73^). Complementary, the DESeq2^69^ R package was used to test for differential genera abundances between the presence or absence of behavior indicators. Next to the time and group factors, the horse dependency was included in the generalized linear model. The *p*-values were adjusted for multiple testing using the Benjamini and Hochberg procedure. DESeq2 comparisons were run with the parameters fitType = “parametric” and sfType = “poscounts”.

## Supplementary Information

Supplementary Information 1.

Supplementary Information 2.

Supplementary Information 3.

Supplementary Information 4.

Supplementary Information 5.

Supplementary Information 6.

Supplementary Information 7.

Supplementary Information 8.

## Data Availability

This Targeted Locus Study project has been deposited at DDBJ/EMBL/GenBank under the accession KELW00000000. The version described in this paper is the first version, KELW01000000. The corresponding Bioproject accession number is PRJNA662009 and the BioSamples accession numbers range from SAMN16072951 to SAMN16073174 (https://www.ncbi.nlm.nih.gov/Traces/study/?acc=PRJNA662009).
