# Supplementary material for: Gut microbiota resilience in horse athletes following holidays out to pasture
Source: Sci Rep. 2021 Mar 3;11:5007. doi: 10.1038/s41598-021-84497-y (PMC7930273; doi:10.1038/s41598-021-84497-y)
Supplement: Supplementary file 8 — Supplementary Information 8. [file 41598_2021_84497_MOESM8_ESM.pdf]

Gut microbiota resilience in horse athletes following holidays out to pasture

Núria Mach*, Léa Lansade, David Bars-Cortina, Sophie Dhorne-Pollet, Aline Foury, Marie-Pierre Moisan and Alice Ruet

SUPPLEMENTARY INFORMATION

Supporting figures

Figure S1. Total dry matter intake and estimated daily concentrate intake, as well as daily nutrient consumption of the main macronutrients at each group and time point

(a) Box plot of total dry matter intake (kg DM/day); (b) Box plot of the daily concentrate intake (kg DM/d); (c) Percentage of daily crude protein intake (% of total DM); (d) Percentage of daily fiber intake (% of total DM). In all cases, boxes show median and interquartile range, and whiskers indicate 5th to 95thpercentile. The box color indicates the treatment group: pasture (green) and control (yellow).

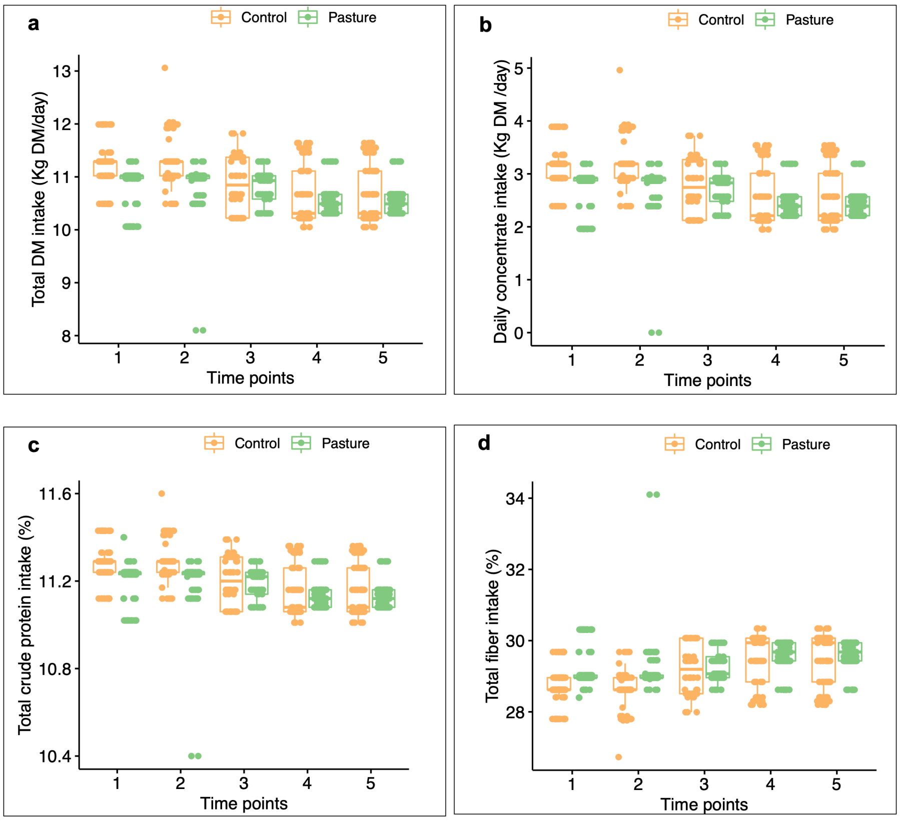

Figure S2. Longitudinal occurrence patterns of diseases for each individual and fecal pH as indicator of gastrointestinal disease

(a) Occurrence patters of disease for each individual at any time point. Each row is an individual. Individuals are split by group (pasture and control). Purple circle indicates the presence of orthopedic injuries while a yellow circle shows the presence of respiratory diseases. Red circle represents digestive problems, green dermatological problems, orange is related to bacterial infection and brown shows equine recurrent uveitis; (b) Relationship between microbiota gut community and fecal pH. Principal coordinate analysis (PCoA) of gut microbiota composition with weighted-UniFrac distance as a function of fecal pH. Fecal pH was categorized based on the calculation of interquartile ranges and standard deviations.

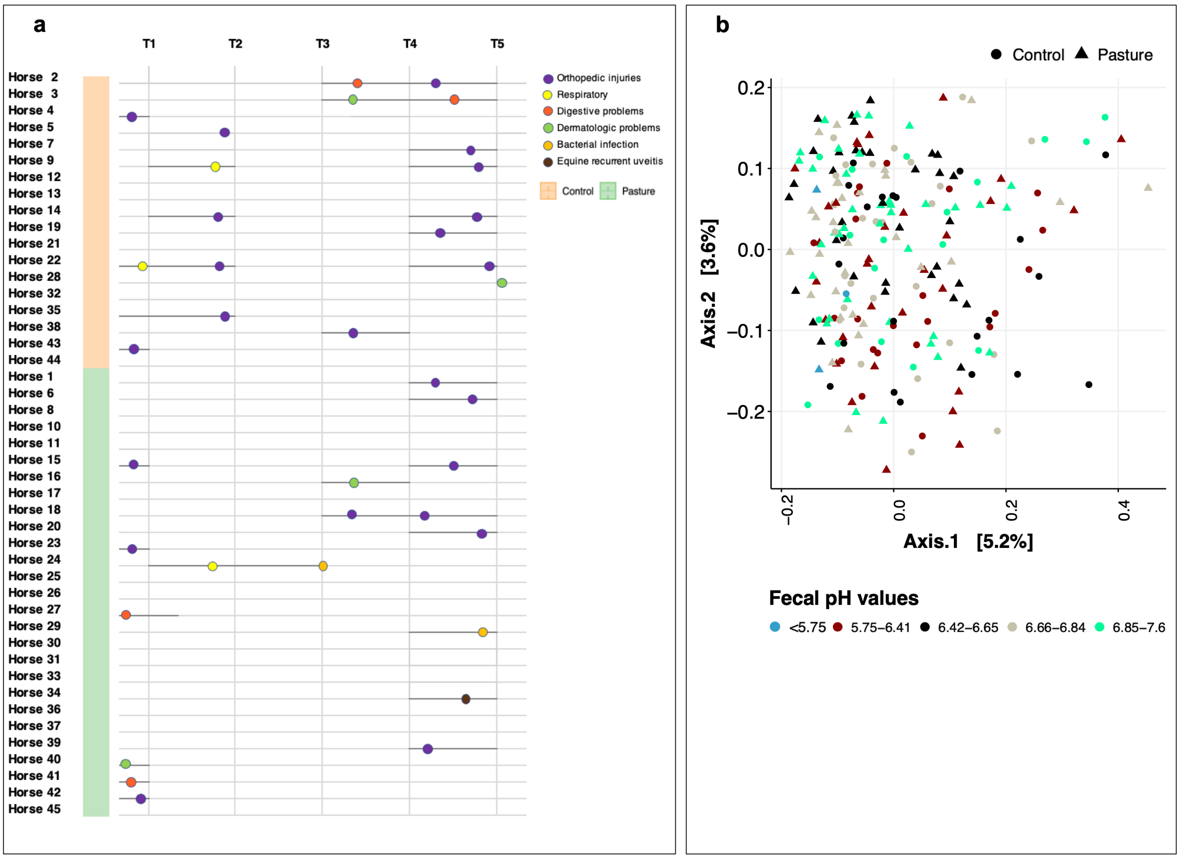

Figure S3. Relationship between microbiota gut community and environmental variables (**a**–**d**) Principal coordinate analysis (PCoA) of gut microbiota composition with weighted-UniFrac distance as a function of performance level, training intensity, disease development and antibiotic administration across the study.

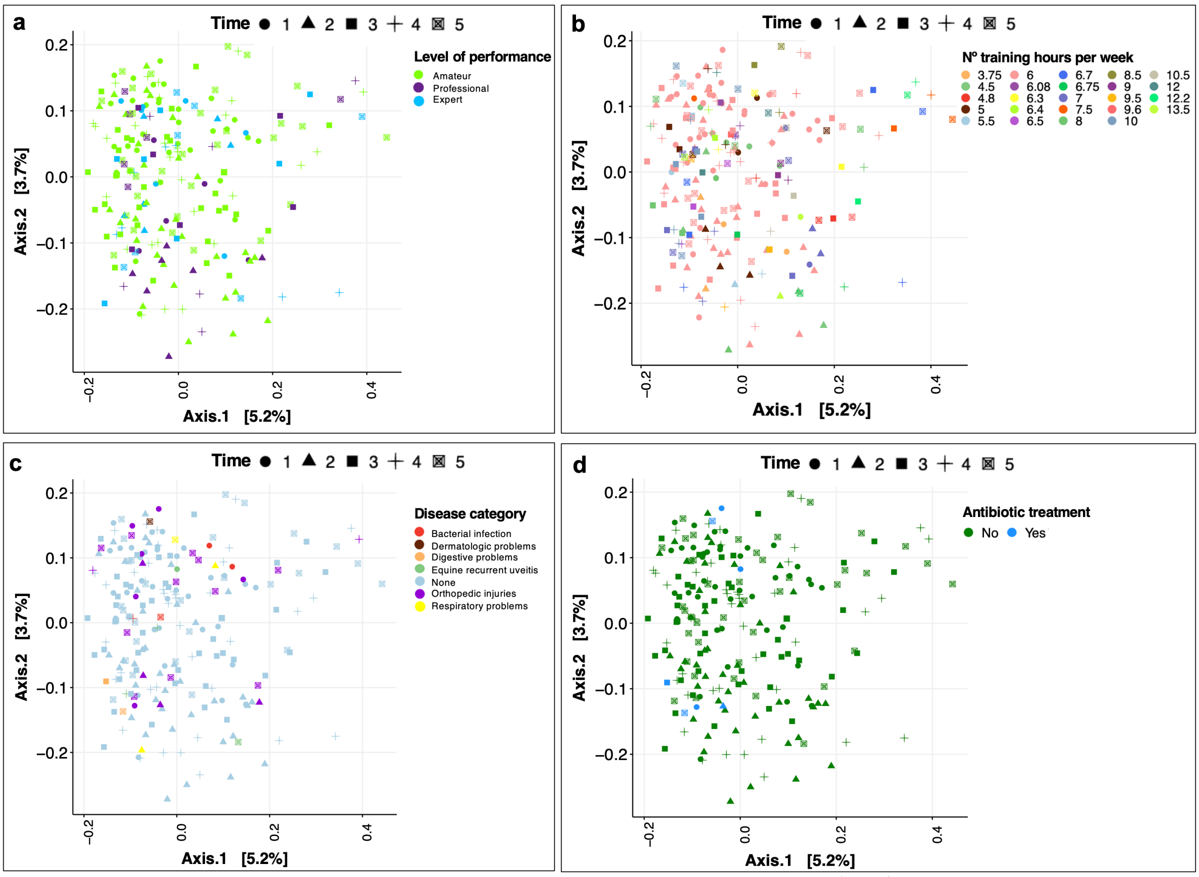

Supporting tables

Table S1. Metadata of the 45 horses recruited in the experiment.

Table S2. Total dry matter intake and estimated daily concentrate and forage intake, as well as daily nutrient consumption for each horse recruited in the experiment. Intakes were recorded from each individual each day of the study and averaged by week or fortnight to reflect habitual dietary intakes.

Table S3. Disease categories and treatments for the 45 horses across the study.

Table S4. The ASVs taxonomical assignments and ASVs counts in each individual and time point of the cohort.

Table S5. Relative abundance (mean ± SD) of each genus at each group and time point.

Table S6. Comparison of differential abundance of microbial genera between the two groups in the fecal microbiota based on the DESeq2 model followed by Benjamini and Hochberg multiple test correction. The table shows the genera that significantly varied in relative abundance between the pasture and control groups, irrespective of the time point, and the information relative to the log fold chance and adjusted *p*-value.

Table S7. Comparison of differential abundance of microbial ASVs between presence or absence of the withdrawn behavior at T5 in fecal microbiota. Differences were calculated between the two situations based on the DESeq2 model followed by Benjamini and Hochberg multiple test correction. The table shows the ASVs that significantly varied in abundance between the presence of withdrawn behavior relative to the absence of the aforementioned behavior at T5. The information relative to the log fold chance and adjusted *p*-value are provided.
